# The Influence of Elevated CO_2_ Concentrations on the Growth of Various Microalgae Strains

**DOI:** 10.3390/plants12132470

**Published:** 2023-06-28

**Authors:** Elizaveta A. Chunzhuk, Anatoly V. Grigorenko, Sophia V. Kiseleva, Nadezhda I. Chernova, Kirill G. Ryndin, Vinod Kumar, Mikhail S. Vlaskin

**Affiliations:** 1 Joint Institute for High Temperatures, Russian Academy of Sciences, 125412 Moscow, Russia; presley1@mail.ru (A.V.G.); k_sophia_v@mail.ru (S.V.K.); chernova_nadegda@mail.ru (N.I.C.); kirillryndin2011@gmail.com (K.G.R.); 2 Faculty of Geography, Lomonosov Moscow State University, 119991 Moscow, Russia; 3 Algal Research and Bioenergy Lab, Department of Food Science and Technology, Graphic Era (Deemed to Be University), Dehradun 248002, India; vinodkdhatwalia@gmail.com; 4 Department of Environmental Safety and Product Quality Management, Peoples’ Friendship University of Russia Named after Patrice Lumumba, 117198 Moscow, Russia

**Keywords:** CO_2_ capture, microalgae, cyanobacteria, high CO_2_ concentrations, microalgae cultivation, *Arthrospira platensis*, *Chlorella ellipsoidea*, *Chlorella vulgaris*, *Gloeotila pulchra*, *Elliptochloris subsphaerica*

## Abstract

The influence of elevated CO_2_ concentrations on the growth and viability of various microalgae strains was studied. *Arthrospira platensis*, *Chlorella ellipsoidea*, *Chlorella vulgaris*, *Gloeotila pulchra*, and *Elliptochloris subsphaerica* were tested. The cultivation of microalgae was carried out at constant CO_2_ concentrations (0.04, 3, 6, or 9%—sequentially from lower to higher concentrations), under constant (24 h·day^−1^) illumination with an intensity of 74.3 µmol quanta·m^−2^·s^−1^, and a constant temperature of 23.5 ± 0.5 °C. The optical density of the microalgae biomass, pH, and the chemical composition of the culture medium were measured. Microscopy (including the cytochemical microscopic method) was conducted to monitor the state of the microalgae. The highest biomass growth rate (0.37 g·L^−1^·day^−1^), among all experiments, was achieved for *Chlorella vulgaris* at CO_2_ = 3% and for *Chlorella ellipsoidea* at CO_2_ = 6 and 9%. The lowest growth rate (0.12 g·L^−1^·day^−1^) was achieved for *Arthrospira platensis* at CO_2_ = 3 and 9%. The microscopy results showed the absence or a minimum number of dead cells of the strains under selected conditions. The ability to maintain the viability of cultures up to significant concentrations of CO_2_ = 9% was due to adaptation (gradual increase in CO_2_ concentrations in the experiments).

## 1. Introduction

In recent decades, there has been a steady increase in the share of renewable energy sources (RESs) in the global fuel and energy balance. One of the goals of such a change is to reduce greenhouse gas emissions into the atmosphere, since about 75% of such emissions come from the energy sector [1].

In addition to the increasing use of RESs, various measures are proposed to reduce greenhouse gas emissions from plants and installations of traditional energy on hydrocarbon fuels. There are various ways to capture and store carbon dioxide (CO_2_), such as pumping gas into underground storage facilities. This method of CO_2_ utilization has several difficulties, primarily related to the volume of underground storage facilities and financial costs. Another way of CO_2_ capture is based on the use of highly efficient photosynthetic organisms [2,3,4].

Currently, the biotechnological potential of microalgae and cyanobacteria is attracting increasing attention from researchers, biotechnologists, and ecologists. It is primarily because microalgae and cyanobacteria are promising raw materials to produce biofuels and valuable related products [5]. Microalgae and cyanobacteria are also used to protect the environment, since, in the photosynthesis process, they convert carbon dioxide into valuable biological products and energy, with much greater efficiency than higher plants, while also releasing oxygen. Due to the simple structure and affordable nutrition, microalgae have theoretically higher photosynthesis efficiency compared to terrestrial plants [6,7]. Algae cultivation does not require arable land, and using wastewater as a nutrient medium will reduce the need for water by 90% and fully satisfy the need for nutrients [8,9,10,11]. On the other hand, the observed active flowering of cyanobacteria in the natural environment over recent decades, associated with an increased CO_2_ content and temperature, requires additional studies, since this can bring substantial impacts on water quality and ecosystem function as whole [12,13]. Additionally, microalgae have a wide range of commercial applications in aquaculture, bioenergy, etc. [14,15].

To achieve economic profitability in obtaining target products from microalgae, a search is underway for both the most productive strains, including genetically modified ones, and the conditions under which this productivity is achieved. In recent years, it has been found that with the joint cultivation of microalgae and bacteria, high growth rates can be achieved [16,17]. Other approaches to finding productive solutions based on consortia of microalgae with other organisms are also being developed. Thus, it is shown that the joint cultivation of microalgae with mycelial fungal spores [18,19], as well as their cultivation on films (the algal biofilm technique method), can significantly speed up the process of biomass collection and reduce costs.

Microalgae plantations or carbon polygons serve as an effective drain of anthropogenic CO_2_, and the removal of cultivated microalgae biomass for energy needs does not violate the natural conservation of organic matter in the biosphere; therefore, algae technologies have recently been considered among existing strategies for CO_2_ capture and storage (CCS) [20].

The exhaust gases of thermal power plants are characterized by quite high concentrations of CO_2_. Therefore, for the effective uptake of CO_2_ by microalgae, it is necessary to search for and adapt the microalgae to obtain strains resistant to high concentrations of CO_2_. It is known that the use of gas mixtures with a high CO_2_ content can increase the specific growth rate of microalgae [21]. At the same time, high concentrations of CO_2_ can be inhibitory for microalgae growth and CO_2_ biofixation [22,23]. Thus, in addition to the search for the most resistant strains of microalgae, it is necessary to determine the most optimal conditions for their cultivation, namely, the maximum concentrations of CO_2_ in gas mixtures at which the high productivity of microalgae is maintained, and, consequently, the biofixation of CO_2_.

To date, studies have been conducted on various strains of microalgae when exposed to high concentrations of CO_2_. In their article, Ambreen Aslam et al. [21] studied the features of CO_2_ absorption and other components in the composition of the flue gases from a coal-fired power plant with a capacity of 4 MW. Preliminary experiments on the cultivation of several highly productive strains of microalgae (e.g., *Scenedesmus dimorphus NT8c*, *Chlorella vulgaris*, *Limnothrix planctonica*) showed that the addition of small amounts of CO_2_ to the gas–air mixture led to almost triple the productivity of biomass compared to growing only in the air under conditions of limited CO_2_ content. To improve the viability and tolerance of microalgae to high concentrations of CO_2_, slow adaptation was required, starting with low CO_2_ concentrations = 1.1% and 3.3%. The final indicator of the survival of microalgae was their high productivity (in terms of biomass), which is associated with the intensity of CO_2_ absorption.

The intensity of CO_2_ absorption obtained in experiments with microalgae directly depends on the choice of strains. In their article, Liyana Yahya et al. [24] assess the ability of microalgae strains *Nannochloropsis* sp., *Tetraselmis* sp., and *Isochrysis* sp. to capture CO_2_, strains were first grown in the laboratory with 100% CO_2_, and then on the flue gases of a coal-fired power plant (Malaysia). The best results after the adaptation phase were shown by *Isochrysis* sp., with a maximum fixation rate (absorption) of 0.35 g·CO_2_·L^−1^ of culture liquid per day. According to [21], the microalgae strain *Desmodesmus* sp. withstood CO_2_ concentrations of up to 11%, while *Spirulina platensis* (or *Arthrospira platensis)* in consortium with other microalgae showed steady growth on a gas–air mixture with a concentration of CO_2_ up to 15% [25]. Giuseppe Olivieri et al. [26] presented the results of the effect of high concentrations of CO_2_ on the growth of microalgae *Stichococcus bacillaris* (ACUF 158/11). It was shown that the concentrations of CO_2_ higher than in the air (0.035% by volume) improved the productivity of microalgae. The same culture of microalgae, *Stichococcus bacillaris*, was subjected to bubbling at high concentrations of CO_2_ in the gas–air mixture (5 and 15%) [27]. In a detailed review of the methods and results of cultivation of a wide range of microalgae [28], *Chlorella* sp. is indicated as a culture that is grown with high efficiency when bubbling with gas–air mixtures with high concentrations of CO_2_. Baohua Zhu et al. [29] studied *Spirulina* strains (LAMB171, LAMB172, and LAMB220) from the Laboratory of Applied Microalgal Biology, Ocean University of China. CO_2_ concentrations were 2, 5, 10, and 15%, and cultivation lasted 12 days. The best biomass performance and the highest CO_2_ fixation rate were obtained at CO_2_ concentrations of 10% for all three strains. The maximum biomass yield was 272.12 mg·L^−1^ per day for the *Spirulina* LAMB171 strain. More results about the tolerance of *Spirulina* to various high CO_2_ concentrations can be found in [30,31,32,33,34,35].

Studies show that, even within the same culture, the productivity of microalgae can significantly differ. A.M. Lizzul et al. [36] conducted experiments with *Chlorella*, and an increase in biomass and lipid production was observed with the addition of CO_2_. *Chlorella* showed satisfactory growth at CO_2_ concentrations both in the range of 1% to 18% by volume [37] and under extreme conditions, with CO_2_ concentrations up to 50% [38]. However, for example [22], there was a significant decrease in the growth of *Chlorella* sp. cells at high CO_2_ concentrations.

The results listed above show that a wide range of microalgae strains have already been considered, for which different results have been obtained, which are not always consistent with each other. In studies for the same culture, both stable productivity at high CO_2_ concentrations and inhibition of microalgae growth were found. This indicates that it is necessary to continue and expand the study of microalgae strains to have the most complete material based on the results of experiments, allowing the selection of the optimal conditions for growing cultures, and the method of adaptation of microalgae strains to high CO_2_ concentrations.

It should also be noted that in previous studies, experiments on the effect of elevated CO_2_ concentrations on the growth and development of microalgae were not carried out in a long multistage adaptation algorithm.

The aim of this study was to investigate various strains of microalgae to identify the most viable and resistant strains when cultivated in a gas–air environment with high CO_2_ concentrations. In our work, we proposed to conduct studies of various strains of green algae and cyanobacteria in one experiment with the same conditions of illumination, temperature, bubbling intensity, and concentration of CO_2_ in the gas–air mixture for a detailed comparative analysis. Both microalgae previously studied for tolerance to high CO_2_ concentrations (*Chlorella vulgaris*, *Chlorella ellipsoidea*, and *Arthrospira platensis*) and newly isolated strains of microalgae, whose studies are rare and can give interesting new results (*Gloeotila pulchra* and *Elliptochloris subsphaerica*), were selected as objects of research.

Since the experiments are focused on the further practical application of the results in large installations for the disposal of CO_2_ from flue gases, it is essential to select and test the methodology of rapid analysis of the viability of microalgae under stressful conditions for CO_2_ concentrations, which do not require lengthy microbiological procedures, which is also the novelty of this study.

Thus, the objectives of the present study were as follows:

– Selection of microalgae strains resistant to high CO_2_ concentrations.
– The cultivation of selected strains at different CO_2_ concentrations (0.04, 3, 6, 9%) and implementation of gradual laboratory adaptation of microalgae to high CO_2_ content over a long period of time (48 days of the experiment).
– Assessment of the microalgae viability according to the state of cell permeability, based on cytochemical staining of living and dead cells with methylene blue dye, followed by cell control ordinary light microscopy.

## 2. Results and Discussions

### 2.1. Biomass Growth Rate

**Figure 1:**
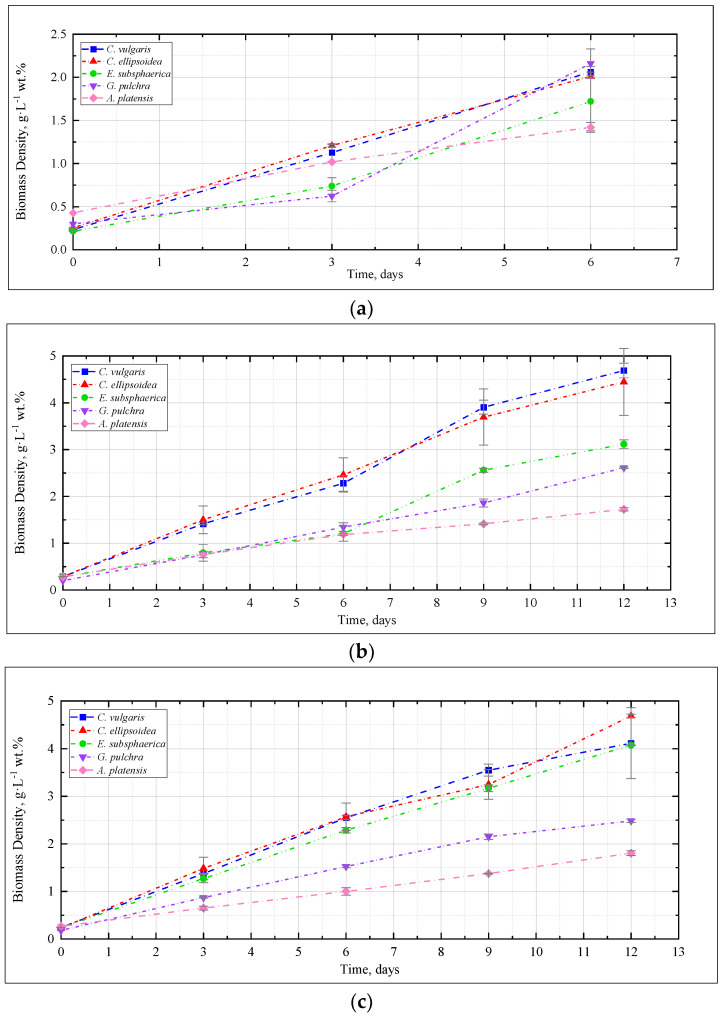

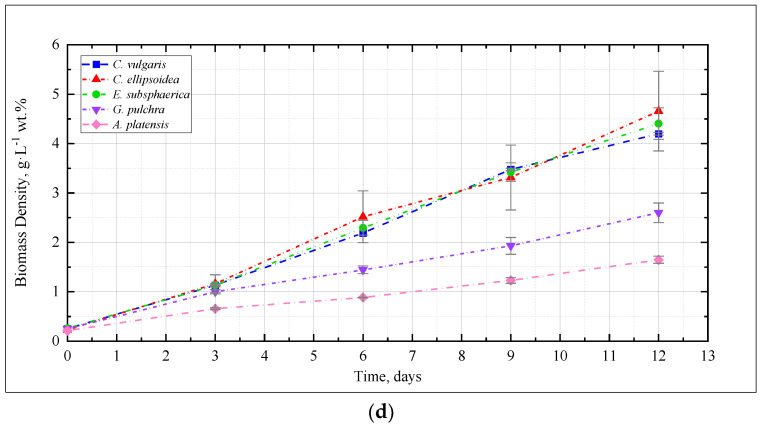
Dynamics of microalgae biomass density in experiments with CO_2_ concentrations: (**a**) 0.04%, (**b**) 3%, (**c**) 6%, (**d**) 9%.

**Figure 2:**
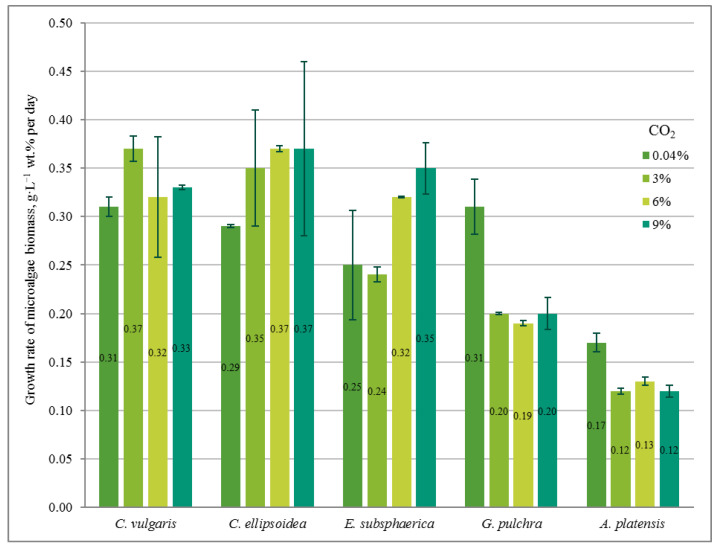
The growth rate of microalgae biomass (g·L^−1^ wt. % per day) based on the results of the experiments.

Figure 1 shows the change in the density of microalgae biomass in the experiments with CO_2_ concentrations of 0.04, 3, 6, and 9%. Figure 2 shows the results of determining the growth rate of microalgae biomass for all experiments.

The dynamics of microalgae biomass density in the experiment with CO_2_ = 0.04% is shown in Figure 1a. Here and further, the mean values for two repetitions and the error (standard deviation) are presented. Throughout the experiment, the microalgae biomass grows steadily. A marked increase in growth rate was observed for *G. pulchra* and *E. subsphaerica* after three days of the experiment.

In the experiment with CO_2_ = 3%, similar to the experiment with CO_2_ = 0.04%, a stable increase in the density of microalgae biomass was observed (Figure 1b). It can be noted that the growth is steady; therefore, there is no significant inhibition of culture growth due to the increased content of CO_2_ in the gas–air mixture. The growth rates of *Chlorella* cultures increased compared to the experiment with CO_2_ = 0.04%, while the growth rate of the strains of *G. pulchra* and *A. platensis* decreased markedly (Figure 2). The biomass growth rate for the *E. subsphaerica* strain remained almost unchanged (Figure 2).

As in the experiments with CO_2_ concentrations of 0.04 and 3%, in the experiment with CO_2_ = 6%, a steady increase in the density of microalgae biomass is observed (Figure 1c). Some tendency of slowing growth is visible only in the last three days of the experiment for *G. pulchra.* The results obtained indicate there is no significant inhibition of culture growth due to the increased CO_2_ content in the gas–air mixture. The maximum growth rate of biomass is achieved for the strain *C. ellipsoidea* (0.37 g·L^−1^ wt. % per day), while, compared with CO_2_ = 3%, the growth rate for the strain *E. subsphaerica* increased markedly; the strains *G. pulchra* and *A. platensis* remained almost unchanged, and the strain *C. ellipsoidea* decreased markedly (Figure 2).

In the experiment with CO_2_ = 9%, it is worth noting the preservation of the steady growth of the biomass density (Figure 1d), as in experiments with CO_2_ concentrations of 0.04, 3, and 6%. There is no inhibition of biomass growth due to an increase in the concentrations of CO_2_ in the gas–air mixture. The maximum growth rate of biomass is maintained for the strain *C. ellipsoidea* (0.37 g·L^−1^ wt. % per day), while, in comparison with the experiment with CO_2_ = 6%, the growth rate for the strain *E. subsphaerica* increased, and strains *C. ellipsoidea*, *G. pulchra*, and *A. platensis* remained almost unchanged (Figure 2).

Thus, for *C. vulgaris*, the highest biomass growth rate was achieved in the experiment with CO_2_ = 3%, for *C. ellipsoidea* with CO_2_ = 6 and 9%, for *E. subsphaerica* with CO_2_ = 9%, and for strains *G. pulchra* and *A. platensis* with CO_2_ = 0.04%.

The results obtained in the experiments on biomass growth rate have been compared with a study similar in goals, tools, and methods [39], where *Chlorella sorokiniana* IPPAS C-1 was considered an object of research. Cultivation was carried out in flat-panel photobioreactors with volumes of 5 and 18 L, lighting was provided by an LED system with an intensity of 900 µmol quanta·m^−2^·s^−1^, and the temperature 35.5 ± 0.5 °C. The productivity of *Chlorella sorokiniana*, depending on CO_2_ concentrations, and the ventilation coefficient of the gas–air mixture were estimated. The CO_2_ content in the gas–air mixture was lower than in our experiments (from 1 to 4%), and the results of the growth rate are presented only for the first three days of the experiment. However, the achieved biomass growth rates were more significant than in our experiments: the maximum growth rate was obtained at a concentration of CO_2_ = 1.5%, and was 1.51 ± 0.07 g·L^−1^ wt. % per day. As a result, after three days in [39], biomass density was achieved in the range of 4–5 g·L^−1^ wt. % at CO_2_ concentrations of 1.5, 2, and 4%. In our experiments, *Chlorella* showed the highest growth among all strains, the rate of which was at a maximum of 0.37 g·L^−1^ wt. % per day. At the same time, there was no tendency to decrease the growth rate with an increase in CO_2_ concentrations up to 9%; the increase in biomass density was stable and constant during the 12 days of the experiment. A comparison of the results suggests the possibility of increasing the growth rate of *Chlorella* microalgae biomass (and, consequently, the intensity of CO_2_ absorption) while optimizing the cultivation regime. In this case, an increase in the illumination intensity can be decisive (in our experiments, the illumination intensity was 74.3 µmol quanta·m^−2^·s^−1^), as well as optimal CO_2_ flow rate. The results also show the importance of the microalgae adaptation procedure to high CO_2_ concentrations, since it increases the efficiency of carbon dioxide absorption. Similar studies were carried out by Zhang B. et al. [40], who showed that adaptation improves the phenotype of microalgae based on random mutation and natural selection.

In our experiments, the microalgae demonstrated different responses to elevated CO_2_ concentrations and the rate of adaptation to these concentrations. Thus, *C. vulgaris* showed an almost constant growth rate at all CO_2_ values, *C. ellipsoidea* and *E. subsphaerica* achieved a significant increase in growth rate with increasing CO_2_, while *G. pulchra* and *A. platensis* were characterized by a decrease in growth rate at CO_2_ = 3, 6, and 9% compared to air. At the same time, the maximum growth rate reached values of the order of 0.35–0.37 g·L^−1^ wt. % per day. This value is consistent with the results obtained in [21]; however, in this work, microalgae consortia demonstrated maximum productivity only at CO_2_ = 1%, without significant changes with increasing CO_2_ content in the gas–air mixture.

The reason for such reaction differences may be the variety of ways of carbon assimilation and sources of carbon in the nutrient media. Thus, for *A. platensis*, the source of carbon is dissolved NaHCO_3_, while for the rest of the microalgae, it is CO_2_ supplied in gaseous form.

### 2.2. pH of the Culture Medium

**Figure 3:**
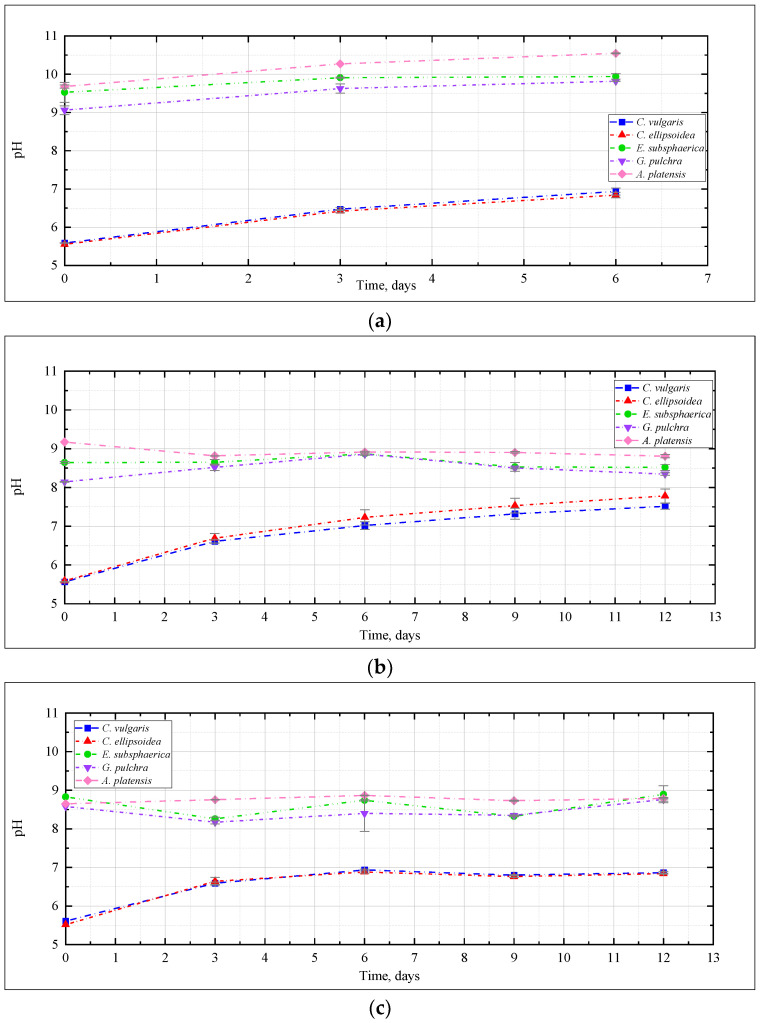

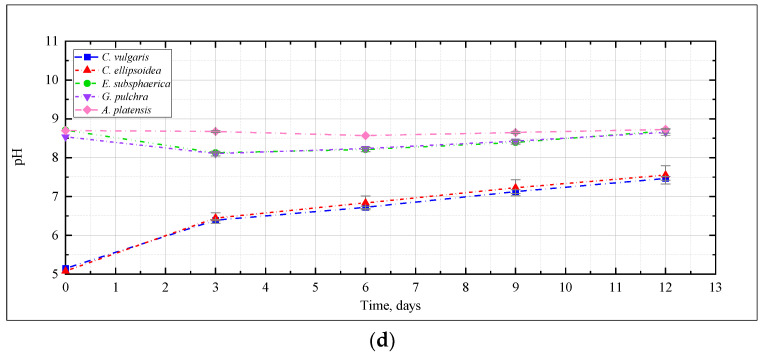
Change of pH during the experiments with CO_2_ concentrations: (**a**) 0.04%, (**b**) 3%, (**c**) 6%, (**d**) 9%.

The change in pH of the culture medium, as one of the most significant characteristics of the conditions for the cultivation of microalgae, is presented in Figure 3. Here and further, the graphs show the mean values for two repetitions and the error (standard deviation). In the experiment with CO_2_ = 0.04% (Figure 3a), a slight increase in the pH value was recorded for the strains *E. subsphaerica* and *G. pulchra*. For the strains *Chlorella* and *A. platensis*, the pH increase was more significant—5.5–5.6 to 6.8–6.9, and from 9.7 to 10.5, respectively.

The change of pH in the experiment with CO_2_ = 3% (Figure 3b) depended on the strain, and generally turned out to be more significant than with CO_2_ = 0.04%. For *Chlorella* strains, a significant increase in pH was recorded from 5.5 to 7.5, and even 8. For the remaining strains of microalgae, the pH remained mostly constant or decreased, as in the case of *A. platensis*, whose pH value decreased from 9.2 to 8.8, which is not particularly typical for this culture. Many publications [21,31,41] show that CO_2_ bubbling causes acidification of media during the cultivation of a wide range of microalgae, even with buffer solutions used to stabilize the pH of the medium.

It should be noted that in the experiment with CO_2_ = 6% for *Chlorella* strains, the change in the pH of culture mediums turned out to be significant: initially, there was a significant increase in pH during the first three days of the experiment, after which this indicator reached a constant value of about 6.8–6.9 (Figure 3c).

In the experiment with CO_2_ = 9%, the change in pH was significant for *Chlorella* strains, and was characterized by a constant increase throughout the experiment. For the remaining strains, an almost constant pH value was observed again, which indicates a slight effect of high CO_2_ concentrations on the pH of the culture medium (Figure 3d).

**Figure 4:**
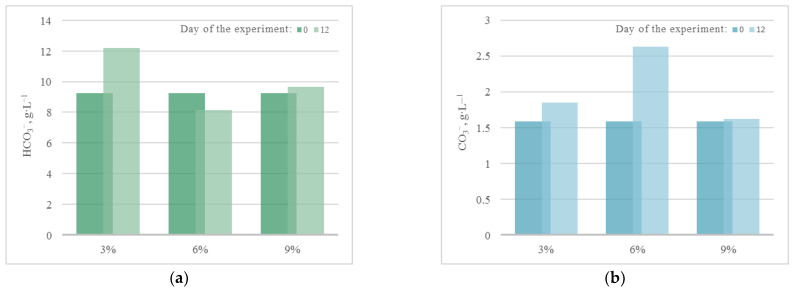
Change in the content of (**a**) bicarbonates and (**b**) carbonates in the medium during cultivation of *A. platensis*.

Thus, for all microalgae strains under these experimental conditions, there was no noticeable acidification of the culture medium due to bubbling with a gas–air mixture with a high CO_2_ content (up to 9%). A constant or slight decrease in pH in experiments with *A. platensis* leads to an increase in the availability of bicarbonates (HCO_3_^−^) for microalgae and more efficient use of the components of the culture medium. The dynamics of the content of bicarbonates and carbonates in the cultivation medium of *A. platensis* in experiments at different CO_2_ concentrations (Figure 4) confirms this statement. It is possible to see a constant, or even slightly increasing, content of HCO_3_^−^ due to the supply of gaseous CO_2_, and an almost constant and low content of carbonates.

Our experiments have shown that pH depends on several factors. First, on the composition of the nutrient medium. Zarrouk’s medium has a significant buffering capacity. Tamiya nutrient medium is unstable, since in the growth process of microalgae, the predominant physiological absorption of NO_3_^−^ anions occurs compared to Na^+^ cations, which leads to an increase in pH. Secondly, in the growth process, CO_2_ bubbling acidifies the nutrient medium to a certain extent, which is confirmed by our experiments. It should be noted that in experiments on the use of flue gases, a decrease in pH during the growth of microalgae is observed [42].

### 2.3. Chemical Composition of the Culture Medium

**Figure 5:**
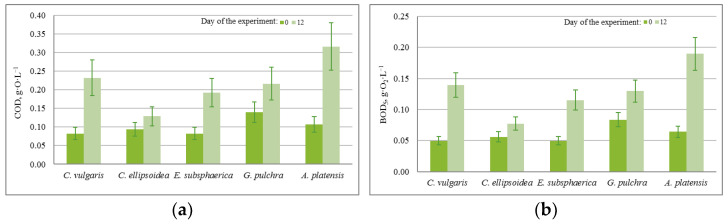
Change in (**a**) chemical oxygen demand (g·O·L^−1^) and (**b**) biological oxygen demand (g·O_2_·L^−1^) with a CO_2_ concentration of 3%.

The results of quantitative chemical analysis (QCA) in the experiment with CO_2_ = 3% showed that during the 12 days of the experiment, there was a significant increase in chemical and biological oxygen demand (COD, BOD_5_), as shown in Figure 5. The growth of COD and BOD_5_ shows that, along with the growth of microalgae, cell death and decomposition occur, accompanied by chemical and biological absorption of oxygen.

**Figure 6:**
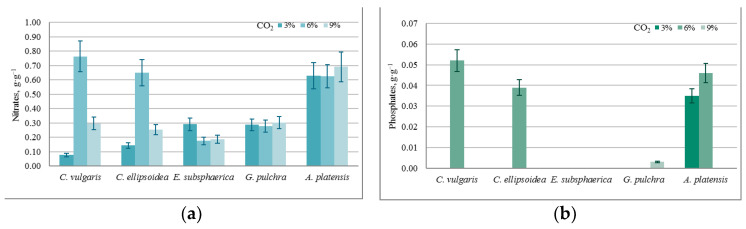
A specific change in the content of culture media components, normalized for biomass growth (g·g^−1^): (**a**) nitrates, (**b**) phosphates.

It should be noted that during the experiment, in all PBRs there was an increase in the concentration of bicarbonates (HCO_3_^−^) in the culture medium due to its constant bubbling with CO_2_. At the same time, an increase in the concentration of carbonates (CO_3_^−^) occurred only in the PBRs with *A. platensis*, which is associated with peculiar properties of the metabolism of this culture. For the rest of the significant components of the culture medium used as nutrients, the following dynamics were found, which are presented in Figure 6.

The almost constant level of phosphate content in the medium with a nonzero increase in biomass in most experiments can be associated with the activity of heterotrophic bacteria, since the experiments were conducted not with axenic, but with algological pure cultures. This assumption is confirmed by the BOD_5_ values obtained in the experiments, which increased significantly during each experiment for all microalgae strains (on average from 0.05 g·O_2_·L^−1^ to 0.190 g·O_2_·L^−1^). In the case of strains *E. subsphaerica* and *G. pulchra*, it is difficult to monitor the dynamics of the phosphate content in the culture medium due to their initial low concentration.

Against the background of a steady increase in biomass, the results of QCA indicate that under the given experimental conditions (CO_2_ = 3%), there is no significant drop in the concentration for components of the culture medium, and, therefore, there is no inhibition of growth.

According to the results of QCA at CO_2_ = 6%, the pattern of the changes in COD and BOD_5_ is the same as in the experiment with CO_2_ = 3%. The change in the concentration of carbonates is also similar to the experiment with CO_2_ = 3%, where the concentration of bicarbonates increased in all cases except the strain *A. platensis*. Thus, in this experiment, significant changes in the content of nutrients were recorded compared to the experiment with CO_2_ = 3%.

According to the results of QCA, in the experiment with CO_2_ = 9%, there is a slight increase in COD and BOD_5_ for strains *Chlorella* and *A. platensis*, while for strains *E. subsphaerica* and *G. pulchra*, the values of these indicators remained unchanged within the margin of error. The change in the concentration of bicarbonates is similar to the experiment with CO_2_ = 6%. For strain *A. platensis*, the concentrations of HCO_3_^−^ and CO_3_^−^ did not change within the margin of error. Thus, in this experiment, significant changes in the content of nutrients were recorded in comparison with experiments with CO_2_ = 3 and 6%.

The obtained QCA results for all of the experiments make it possible to evaluate the intensity of consumption of individual components of culture medium, as well as to determine the mode of adding nutrients to maintain high productivity during long-term cultivation of microalgae.

### 2.4. Results of Microscopic Analysis of Microalgae

**Figure 7:**
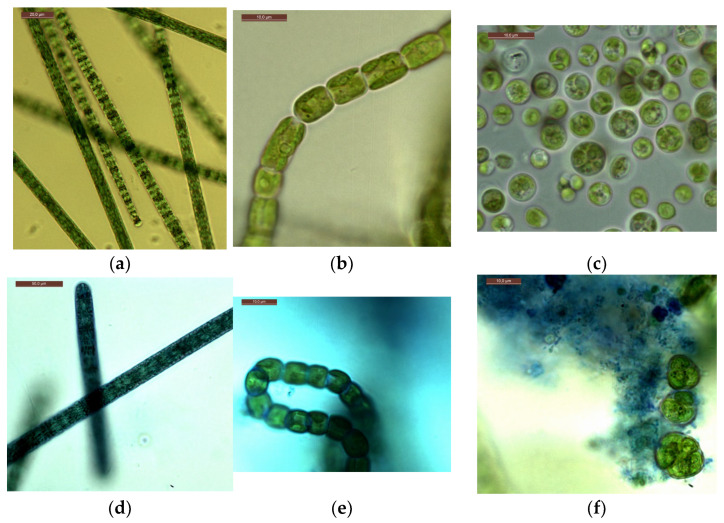
Photomicrographs: *A. platensis*—(**a**,**d**); *G. pulchra—*(**b**,**e**); *E. subsphaerica—*(**c**,**f**). Trichomes and cells without staining: (**a**–**c**), trichomes and cells with staining: (**d**–**f**). Concentration of CO_2_ = 3%, sixth day of the experiment.

Microscopy of strains grown at CO_2_ = 3% showed that, visually, the cells of microalgae did not differ in morphometric parameters from the cells of culture samples from experiments with a CO_2_ content of 0.04% (air), as well as from cells from the working collection of algae of the Renewable Source Energy Laboratory at Lomonosov Moscow State University (RSE LMSU). The results of intravital staining of microalgae strains showed the absence or a minimal number of dead cells. Examples of photomicrographs are shown in Figure 7. They show the integrity of the microalgal cell walls. In the case of *A. platensis* and *G. pulchra*, only the mucous membranes of the microalgal trichomes are stained (Figure 7d,e), but not the internal parts.

Similar results were obtained when growing strains with CO_2_ = 6%. Thus, almost the entire mass of cells of microalgae strains grown at high CO_2_ concentrations (3%, 6%) remained alive during the experiments, which indicates the preservation of the viability of cultures.

**Figure 8:**
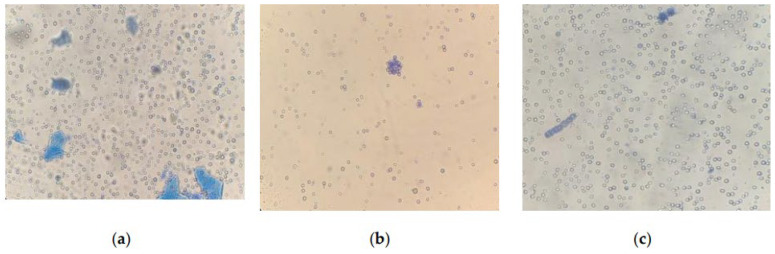

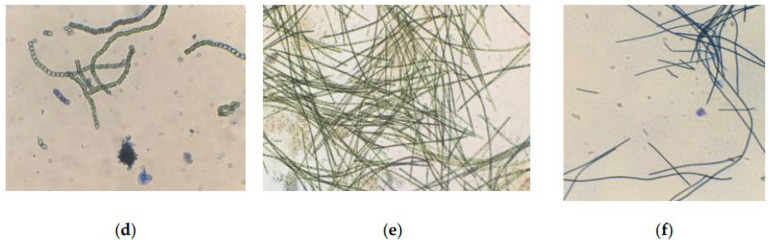
Cells of microalgae strains: (**a**) *C. vulgaris*, (**b**) *C. ellipsoidea*, (**c**) *E. subsphaerica*, (**d**) *G. pulchra*. Samples with staining, magnification ×400; (**e**) *A. platensis*, sample without staining, ×100; (**f**) *A. platensis*, sample with staining, ×100. CO_2_ = 9%, 12th day of the experiment.

Microscopy of strains grown for 6 days at CO_2_ = 9% showed that the cells were not morphologically modified. When staining living cells, only single dead cells stained with methylene blue are found in the samples. However, the results obtained on the 12th day showed insignificant, but noticeable changes in the state of the microalgae (Figure 8).

An analysis of photomicrographs of strains (Figure 8a–d) shows that the main mass of cells in the field of view are alive (unstained), against which clusters of dead colored cells are visible, the proportion of which is low. In the photo of *E. subsphaerica*, a strand of microalga, *G. pulchra*, is visible, which, as a result of contamination, was in small quantities already on the sixth day of the experiment. One strand of *G. pulchra* is colored (dead), which indicates the displacement of this strain by the main strain *E. subsphaerica.* In the photo of the strain *G. pulchra* (Figure 8d), in addition to living unstained cells, blue-colored remains of dead cells are visible in the form of separate short strands. The living strands of *G. pulchra* are long and curved, which indicates unfavorable conditions for the growth of this culture. In Figure 8e,f, *A. platensis* trichomes are shown, mostly alive (only the mucous membrane of cells is stained) and very long; a small number of short trichomes are visible in the field of view, which indicates a weakening of the process of cell growth and division. There are accumulations of detritus. Uncharacteristic convoluted trichomes of this strain are noted.

Thus, by the 12th day of the experiment, at CO_2_ = 9%, signs of some gradual inhibition of cells are detected, which are expressed as deviations in the shape of cells from normal, elongation (lack of division), and an increase in the number of dead cells. At the same time, mass death is not observed, even with such significant CO_2_ content in the gas–air mixture. In earlier work [35], where the strain *A. platensis* was grown at high CO_2_ concentrations under similar conditions, sustained growth could not be achieved: at a high concentration of CO_2_ (9%), mass cell death occurred. Based on the results of the current work, it can be concluded that the optimal method for adapting microalgae strains to high CO_2_ concentrations was selected and used.

## 3. Materials and Methods

### 3.1. Microalgae Strains and Nutrient Medium

During the initial screening, cultures of microalgae and their consortia with bacteria were selected. The following strains of microalgae with a high growth rate and capable of responding to changing environmental conditions, such as a decrease/increase in the concentration of the main nutrient elements, lability with respect to toxic elements, etc., [14] from the collection of the Renewable Source Energy Laboratory at Lomonosov Moscow State University (RSE LMSU), were used for the experiments: *Chlorella vulgaris rsemsu Chv-20/11-Ps*, *Chlorella ellipsoidea rsemsu Chl-el*, *Gloeotila pulchra rsemsu Pz-6*, *Elliptochloris subsphaerica rsemsu N-1/11-B*, and a stable consortium of microalgae/cyanobacteria *Arthrospira platensis rsemsu P Bios* with heterotrophic bacteria (heterotrophic bacteria are the representatives of the genera *Pseudomonas*, *Bacterium*, and *Bacillus*).

The following are descriptions of cultures and nutrient media, as well as the rationale for choosing the listed microalgae for conducting the experiments with high CO_2_ concentrations.

#### 3.1.1. *Arthrospira platensis*

*Arthrospira platensis* (NCBI: https://www.ncbi.nlm.nih.gov/nuccore/KU855375 accessed on 10 June 2023) is a representative of one of the most well-known and cultivated microalgae. It is a filamentous blue-green microalga (cyanobacterium) with straight trichomes. The advantages of *A. platensis* are, firstly, the ability to grow in open cultivators without contamination by other microorganisms due to the high alkalinity of the nutrient medium for its cultivation (pH = 8.5–11.5), and secondly, the ability to use cheap gravitational methods of biomass harvesting due to the relatively large size of its filaments (up to 800 μm). The microalgae used as primary seeding were grown in planar open-type cultivators with a volume of 500 L, in a semicontinuous way on Zarrouk’s nutrient medium at a constant illumination of 25 ± 3 μE·m^−2^·s^−1^ and a temperature of 21 °C with near-surface mixing. The composition of Zarrouk’s medium for cultivation of *A. platensis* [43]: NaHCO_3_—16.8 g·L^−1^, KNO_3_—3.0 g·L^−1^, K_2_HPO_4_·3H_2_O—0.66 g·L^−1^, K_2_SO_4_—0.5 g·L^−1^, MgSO_4_·7H_2_O—0.2 g·L^−1^, NaCl—1.0 g·L^−1^, CaCl_2_—0.04 g·L^−1^, FeSO_4_·7H_2_O—0.018 g·L^−1^, EDTA—0.08 g·L^−1^, microelement solution for Zarrouk’s medium—1 mL·L^−1^.

#### 3.1.2. *Chlorella ellipsoidea* and *Chlorella vulgaris*

*Chlorella ellipsoidea* Gern and *Chlorella vulgaris* Beijer were obtained in 1990 from the collection of microalgae cultures, the Microbiology Department, Faculty of Biology Lomonosov MSU (*C. ellipsoidea* DMMSU-4, *C. vulgaris* DMMSU-82). *C. ellipsoidea* and *C. vulgaris* are free-living unicellular green microalgae from the genus *Chlorella*. Culture cells are solitary, small, ellipsoid to spherical in shape, with a diameter of 1.3–6.8 μm. *C. ellipsoidea* and *C. vulgaris* were selected according to the results of literature screening [21,28,36,37,38,44] and a preliminary study. These strains are acidophilic; thus, they grow well on Tamiya nutrient medium with the initial pH value of about 5.5. Composition of Tamiya medium (on distilled water) [43]: KNO_3_—5.0 g·L^−1^, KH_2_PO_4_—1.25 g·L^−1^, MgSO_4_·7H_2_O—2.5 g·L^−1^, FeSO_4_·7H_2_O—0.009 g·L^−1^, EDTA—0.037 g·L^−1^, H_3_BO_3_—2.86 mg·L^−1^, MnCI_2_·4H_2_0—1.81 mg·L^−1^, ZnSO_4_·7H_2_O—0.22 mg·L^−1^, MnO_3_—0.018 mg·L^−1^, NH_4_VO_3_—0.023 mg·L^−1^.

#### 3.1.3. *Gloeotila pulchra*

*Gloeotila pulchra* (NCBI: https://www.ncbi.nlm.nih.gov/nuccore/KU961671 accessed on 10 June 2023) is a green, light-loving, free-living planktonic microalgae. The cells are cylindrical, 3.5–4 μm wide and 20–30 μm long, straight, and rounded at the ends. This microalga was selected based on the results of the preliminary screening of cultures. BG-11 cultivation medium composition [43]: NaNO_3_—1.5 g·L^−1^, K_2_HPO_4_·3H_2_O—0.04 g·L^−1^, MgSO_4_·7H_2_O—0.075 g·L^−1^, CaCl_2_·2H_2_O—0.04 g·L^−1^, Na_2_CO_3_—0.02 g·L^−1^, citric acid—0.006 g·L^−1^, Na_2_EDTA—0.001 g·L^−1^, ammonium iron citrate—0.006 g·L^−1^, microelement solution—1 mL·L^−1^. Composition of trace elements: H_3_BO_3_—2.86 g·L^−1^; MnCl_2_·4H_2_O—1.81 g·L^−1^; ZnSO_4_·7H_2_O—0.22 g·L^−1^; Na_2_MnO_4_·2H_2_O—0.4 g·L^−1^; CuSO_4_·5H_2_O—0.08 g·L^−1^; Co(NO_3_)_2_·7H_2_O—0.05 g·L^−1^.

#### 3.1.4. *Elliptochloris subsphaerica*

*Elliptochloris subsphaerica*, (*basionym: Pseudochlorella subsphaerica Reisigl*, 1964) [NCBI: https://www.ncbi.nlm.nih.gov/nuccore/KU926337 accessed on 10 June 2023] is a planktonic unicellular green microalgae. Algae from this genus have round cells with single chloroplasts divided into two parts, and the cytoplasm is very granulated; the presence of a pyrenoid differs from other species of this genus. The cells have a diameter of 4–8 μm, withlarge cells up to 10 μm in size. BG-11 cultivation medium was used to cultivate *E. subsphaerica.*

### 3.2. Experimental Setup

To conduct experiments to test the viability of microalgae strains at high CO_2_ concentrations, a laboratory installation was created, which includes the following main elements: the atmospheric gas chamber (AGC) and photobioreactors (PBRs) (10 pcs).

#### 3.2.1. Atmospheric Gas Chamber (AGC)

An AGC with a control unit was designed to create a controlled atmosphere with a given composition and parameters (temperature, pressure, gas composition, etc.). The internal volume of the chamber is 12 m^3^: width 2 m, length 3 m, height 2 m. The chamber is equipped with a door with a width of 0.6 m and a height of 1.6 m.

The AGC is equipped with a heater, cooler, blower, and sockets. The heater is designed to heat the air inside the chamber, the cooler is designed to cool the air inside the chamber, the blower is designed to mix the air mass inside the chamber, and the sockets are designed to connect electrical equipment inside the chamber. The AGC is also equipped with analytical instrumentation: a pressure sensor, a humidity sensor, contact-type temperature sensors with the ability to install at any point inside the chamber, and a gas composition analyzer. The gas analyzer installed in the chamber can determine the concentration of the following gases: oxygen, carbon monoxide, CO_2_, ammonia, methane, sulfur dioxide, and nitrogen dioxide.

A gas-discharge ramp with CO_2_ cylinders is connected to the chamber. Thus, the AGC can be configured to automatically maintain given CO_2_ concentrations. Additionally, the AGC can be configured to automatically maintain the set temperature inside the chamber. A detailed description of the AGC is presented in the earlier work [35].

#### 3.2.2. Photobioreactors (PBRs)

PBRs for testing the viability of microalgae strains at high CO_2_ concentrations consist of the following main elements:

– Reactors (designed for placing a nutrient medium with microalgae inoculum inside them).
– LED system (designed to evenly distribute the luminous flux around the perimeter of the reactor using strip LED lamps).
– Power supply system of the LED system (designed to supply electrical energy to the LED system), as well as to obtain a luminous flux from the LED system of a certain intensity.
– A system for supplying a gaseous medium from the atmospheric gas chamber to the zone with a suspension of microalgae (designed to ensure continuous bubbling of gas from the atmosphere of the gas chamber through a suspension of microalgae throughout the experiment).

**Figure 9:**
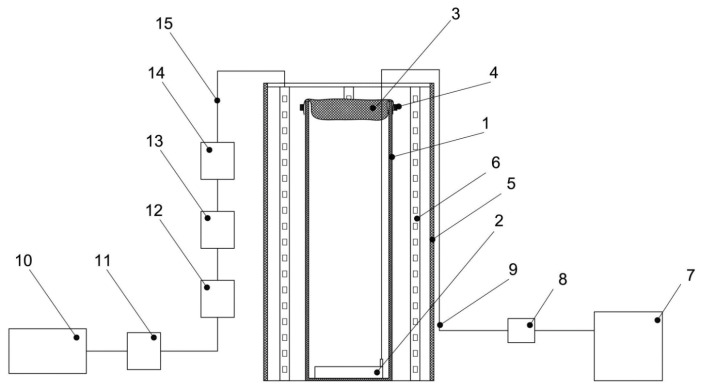
Schematic diagram of PBR: 1—glass flask, 2—air aerator, 3, 4—textile cover for the PBR made of nonsterile cheesecloth, 5—galvanized pipe, 6—LED strip, 7—compressor, 8—check valve, 9—silicon hose, 10—power supply, 11—dimmer, 12–15—terminals for connecting LED strips to the power supply.

**Figure 10:**
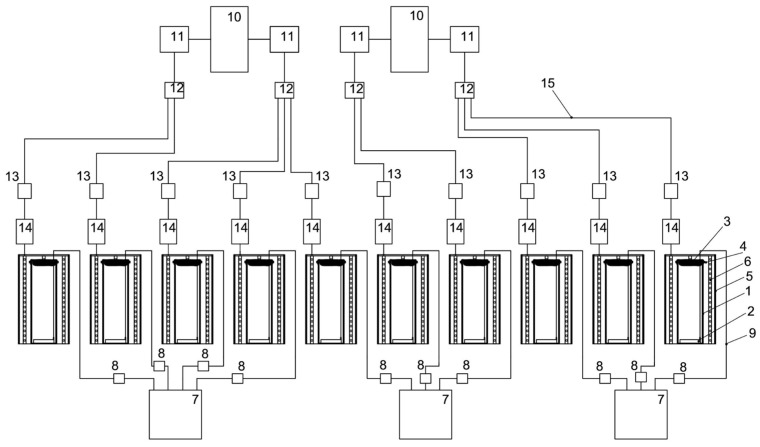
Schematic diagram of ten PBRs involved in the experiments: 1—glass bulb, 2—air aerator, 3—gauze, 4—stationery elastic band, 5—galvanized pipe, 6—LED strip, 7—air compressor, 8—check valve, 9—silicone hose, 10—power supply, 11—dimmer, 12—universal terminal block mounting branching 2 × 6 wires, 13—power connector kit, 14—universal mounting terminal block 2-wire double-sided, 15—power cable.

**Figure 11:**
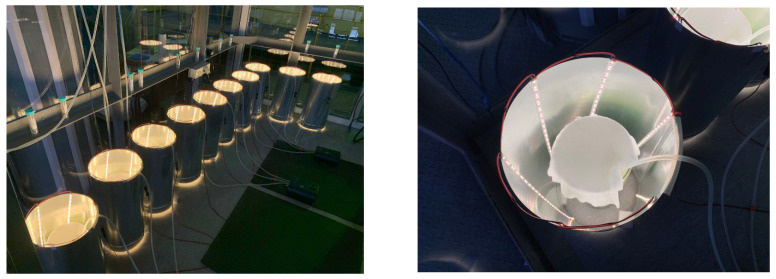
A general view of ten PBRs inside the AGC and a top view of a single PBR.

The main components of the reactor are a cylindrical glass flask with a height of 40 cm, an external diameter of 15 cm, a wall thickness of 3 mm, a textile cover for the PBR made of nonsterile cheesecloth with a size of 0.9 × 0.5 m, and an air aerator with the size of 80 × 50 mm, placed at the bottom of the glass flask. The LED system includes a 0.5 m galvanized pipe, a 3 m long LED strip, and a terminal block. The LED system provided illumination of 74.3 µmol quanta·m^−2^·s^−1^ on the inside of the PBR. Block diagrams on the example of one PBR and the 10 PBRs involved in the experiment are shown in Figure 9 and Figure 10, respectively. A general view of the PBR system in the amount of 10 pieces inside the AGC and a top view of a single PBR are shown in Figure 11.

### 3.3. Experimental Procedure

The experiments were carried out using the PBR in the amount of 10 pieces, placed in the AGC. Uniform illumination was provided around the perimeter and height of the PBR using strip LED lights. Illumination was constant (i.e., 24 h per day). The gas–air mixture was fed to the PBR through air aerators, while the gas flow rate adjusted by the compressor was 1 L·min^−1^. Each reactor was covered with a textile cover on top to minimize contamination of the PBR with nontarget microalgae strains.

Experiments were carried out on the microalgae cultivation on gas–air mixtures with a consequent increase in CO_2_ content: 0.04% (air), 3%, 6%, and 9%. The method description is given in [35,45]. Each experiment was conducted according to the following procedure:

1. Preparation of nutrient medium on distilled water.
2. Seeding the medium with an inoculum of each strain to the initial concentration of microalgae biomass (0.2–0.25) g·L^−1^ in terms of dry weight (wt. %).
3. Filling two PBRs with cultural liquid (nutrient medium with inoculum): 4 L in each PBR for each strain.
4. Placement of PBRs in the AGC, turning on the lighting and bubbling. Illumination is constant during the experiment.
5. CO_2_ injection to a predetermined concentration in the AGC, sealing the chamber.
6. Cultivation of microalgae during 12 days at given constant CO_2_ concentrations with a sampling of a suspension of microalgae on days 0, 3, 6, 9, and 12 of cultivation to determine the density and growth rate of biomass, pH, as well as the content of nutrients.
7. After the end of the experiment and sampling for analysis, microalgae biomass with culture liquid was placed into 5 L containers. This biomass was used as a source of inoculum for seeding of the PBR in the next experiment. The application of this procedure is proposed for the first time.

### 3.4. Research Methods

#### 3.4.1. The Schedule of Sampling and Analyses

The schedule of sampling, analysis of the state of microalgae (optical density of biomass—OD, pH of the medium, and microscopy), and the composition of the culture medium are presented below:

– Composition of the medium—on days 0 and 12.
– Microscopy—on days 0, 6, and 12.
– OD and pH—on days 0, 3, 6, 9, and 12.

#### 3.4.2. The Conditions of the Experiments

The conditions of the experiments were:

– Microalgae strains: *C. vulgaris*, *C. ellipsoidea*, *E. subsphaerica*, *G. pulchra*, *A. platensis*.
– Temperature in the AGC: 23.5 ± 0.5 °C.
– PBR illumination intensity: 74.3 µmol quanta·m^−2^·s^−1^.
– Water: distilled.
– Duration: 6 days (experiment no. 1), 12 days (experiments no. 2–no. 4).
– CO_2_ concentrations: 0.04% (experiment no. 1), 3% (experiment no. 2), 6% (experiment no. 3), and 9% (experiment no. 4).

#### 3.4.3. Nutrient Medium Analysis

The following methods and measuring instruments were used to measure these characteristics:

– OD determination using photometer Expert-003;
– pH determination using the pH meter Expert-pH;
– Quantitative chemical analysis (QCA) was carried out according to the methods presented in Table 1 (the error of the determination methods is about 10%).

#### 3.4.4. Microscopic Analysis

The viability of microorganisms is an integral characteristic of all living systems. This term refers not only to the concept of “living”, but also includes the characteristic, for example, of the genetic and phenotypic usefulness of a living object, its ability to function normally in various, including unfavorable, conditions, as well as the adaptive capabilities of cells, their growth, and reproductive potentials. The paper [46] provides a classification of methods for determining the viability of microorganisms, which are divided into direct and indirect. The first includes methods based on direct consideration of the reproductive ability of living cells, for example, determining the growth rate of biomass. The second—methods of indirect manifestations of cell activity—includes the permeability of the cell wall and the ability of the cell to be stained with various dyes. In the presented work, the method of viability control by the state of the cell permeability barrier based on cytochemical staining of living and dead cells with vital dyes by microscopy and the microculture method according to Imshenetsky were used [47]. The essence of the method of vital staining is that some dyes can penetrate the cell only through the damaged membrane of dead cells, and stain their cytoplasm. Living cells, with undisturbed selective permeability of the cell wall or membrane, do not allow the dye to pass through, and remain colorless. Using methylene blue dye [47,48,49,50], living cells remain colorless, and dead cells are stained blue. Additionally, vital staining of cells with methylene blue makes it possible to assess the metabolism of cells and identify their early changes and, as a result, characterize the state of cells in culture.

Thus, an express method has been developed and tested for determining the viability of microalgae and cyanobacteria cells using a conventional light microscope, in which cell staining occurs/does not occur in a drop of the sample on a sterile slide and cover glasses when adding the intravital aqueous dye methylene blue. As a result, a microcultural method is implemented on a slide in 3–5 min. The method described above was developed for a simple and applicable method for the industrial cultivation of microalgae and cyanobacteria.

In this study, the evaluation of the viability of microalgae and cyanobacteria cells using the cytochemical microscopic method was carried out using the methylene blue aqueous dye at a concentration of 1:10,000. Microscopic monitoring of the state of microalgae cultures and their viability was carried out using a Leica DM 2500 digital microscope and a Mikmed-5—LOMO light microscope. At least 10 fields from each sample of experiments taken on the 6th and 12th day were viewed; at the maximum magnification of the microscope, the number of blue-stained (dead) cells or their clusters was counted, as well as a botanical description of the state of the cells and photography.

## 4. Conclusions

This study is devoted to the important issue of capturing CO_2_ with the help of microalgae, which may be of practical importance in the application to carbon dioxide industrial emissions. Cultivation was carried out under controlled laboratory conditions in photobioreactors placed in a closed atmospheric gas chamber; the growth characteristics of microalgae strains (*C. vulgaris*, *C. ellipsoidea*, *E. subsphaerica*, *G. pulchra*, *A. platensis*) at various CO_2_ concentrations (CO_2_ = 0.04, 3, 6, and 9%) were determined.

To assess the achievable viability of microalgae at high CO_2_ concentrations, a sequential adaptation mode was implemented in the experiments, when the microalgae biomass grown at lower CO_2_ concentrations was used as an inoculum at the next stage of the experiment at higher CO_2_ concentrations. The effectiveness of this approach was confirmed by previous studies with a consortium based on *A. platensis* with heterotrophic bacteria, when the lack of such a gradual adaptation led to the death of *A. platensis* cells under a concentration of 9% CO_2_. Under all concentrations of CO_2_, increasing biomass density of all microalgae strains was observed; however, the growth rate was different, which indicates the individual resistance of strains to excess CO_2_. As the concentration of CO_2_ increased, strains *C. vulgaris*, *C. ellipsoidea*, and *E. subsphaerica* showed a tendency to increase in growth rate (up to 0.35–0.37 g·L^−1^ wt. % per day), while strains *G. pulchra* and *A. platensis*, on the contrary, showed a significant decrease in growth rate (from 0.31 to 0.20 g·L^−1^ wt. % per day in case of *G. pulchra*).

These experiments made it possible to determine the most effective strains that can withstand high concentrations of CO_2_, as well as to show the need for a longer adaptation for such technological and productive strains as *A. platensis*. An express method has been proposed and tested for determining microalgae cell viability using a light microscope based on cytochemical staining of living and dead cells with the vital dye methylene blue. This method made it possible to determine an insignificant proportion of dead cells even at 9% CO_2_ concentration, though some gradual inhibition of cells is revealed, which is expressed in deviations of the cell shape from the norm at higher concentrations of CO_2_.

Thus, effective strains of microalgae have been identified, and the effectiveness of the method used for their adaptation to high CO_2_ concentrations has been shown. These results, as well as the proposed method of express microscopic analysis of the state of microalgae strains, can serve as a pilot plant’s basis for the utilization of carbon dioxide from gas–air mixtures.

## Figures and Tables

**Table 1:** Components of the cultural medium and methods for their determination.

Indicator	Regulation for Measurement Procedure	Determination Method	Instruments, Equipment
COD, mg·O·dm^−3^	PNDF 14.1:2:3.100-97 (ed. 2016)	Titration	–
BOD_5_, mg·O_2_·dm^−3^	PNDF 14.1:2:3:4.123-97 (ed. 2004)		
Bicarbonates, mg·dm^−3^	GOST 31957-2012, method A.2		
Carbonates, mg·dm^−3^			
Phosphates, mg·dm^−3^	GOST 31867-2012, item 4	Ion chromatography and capillary electrophoresis	ICS-1600 ion chromatograph with conductivity detector
Nitrates, mg·dm^−3^			
Sulfates, mg·dm^−3^			

## Data Availability

Not applicable.
